# 2,3-Dibromo-3-(4-chloro­phen­yl)-1-(2-hy­droxy­phen­yl)propan-1-one

**DOI:** 10.1107/S1600536811036798

**Published:** 2011-09-17

**Authors:** Hoong-Kun Fun, Wan-Sin Loh, B. K. Sarojini, V. Musthafa Khaleel, B. Narayana

**Affiliations:** a X-ray Crystallography Unit, School of Physics, Universiti Sains Malaysia, 11800 USM, Penang, Malaysia; b Department of Chemistry, P. A. College of Engineering, Mangalore 574 153, India; c Department of Chemistry, Mangalore University, Mangalagangotri 574 199, India

## Abstract

In the title mol­ecule, C_15_H_11_Br_2_ClO_2_, an *S*(6) ring motif is formed *via* an intra­molecular O—H⋯O hydrogen bond. The dihedral angle formed between the chloro- and hy­droxy-substituted benzene rings is 34.10 (15)°. In the crystal, weak inter­molecular C—H⋯O hydrogen bonds link the mol­ecules into chains along the *c* axis.

## Related literature

For applications of chalcone compounds, see: Liu *et al.* (2003 ▶); Nielson *et al.* (1998 ▶); Rajas *et al.* (2002 ▶); Dinkova-Kostova *et al.* (1998 ▶); Goto *et al.* (1991 ▶); Uchida *et al.* (1998) ▶; Tam *et al.* (1989 ▶); Indira *et al.* (2002 ▶); Sarojini *et al.* (2006 ▶). For related structures, see: Butcher, Yathirajan, Anilkumar *et al.* (2006 ▶); Butcher, Yathirajan, Sarojini *et al.* (2006 ▶); Harrison *et al.* (2005 ▶); Yathirajan, Mayekar *et al.* (2007 ▶); Yathirajan, Vijesh *et al.* (2007 ▶). For hydrogen-bond motifs, see: Bernstein *et al.* (1995 ▶).
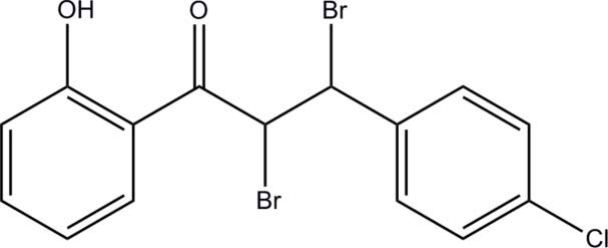


## Experimental

### 

#### Crystal data

C_15_H_11_Br_2_ClO_2_
*M* *_r_* = 418.51
Monoclinic, 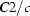

*a* = 29.075 (3) Å
*b* = 9.2358 (10) Å
*c* = 11.4374 (12) Å
β = 103.290 (2)°
*V* = 2989.0 (6) Å^3^
*Z* = 8
Mo *K*α radiation
μ = 5.60 mm^−1^
*T* = 297 K
0.39 × 0.36 × 0.22 mm

#### Data collection

Bruker SMART APEXII DUO CCD area-detector diffractometer
Absorption correction: multi-scan (*SADABS*; Bruker, 2009 ▶) *T*_min_ = 0.218, *T*_max_ = 0.379
16663 measured reflections
5375 independent reflections
3337 reflections with *I* > 2σ(*I*)
*R*_int_ = 0.034

#### Refinement

*R*[*F* ^2^ > 2σ(*F* ^2^)] = 0.037
*wR*(*F* ^2^) = 0.122
*S* = 1.04
5375 reflections
181 parameters
H-atom parameters constrained
Δρ_max_ = 0.61 e Å^−3^
Δρ_min_ = −0.47 e Å^−3^

### 

Data collection: *APEX2* (Bruker, 2009 ▶); cell refinement: *SAINT* (Bruker, 2009 ▶); data reduction: *SAINT*; program(s) used to solve structure: *SHELXTL* (Sheldrick, 2008 ▶); program(s) used to refine structure: *SHELXTL*; molecular graphics: *SHELXTL*; software used to prepare material for publication: *SHELXTL* and *PLATON* (Spek, 2009 ▶).

## Supplementary Material

Crystal structure: contains datablock(s) global, I. DOI: 10.1107/S1600536811036798/lh5331sup1.cif

Structure factors: contains datablock(s) I. DOI: 10.1107/S1600536811036798/lh5331Isup2.hkl

Supplementary material file. DOI: 10.1107/S1600536811036798/lh5331Isup3.cml

Additional supplementary materials:  crystallographic information; 3D view; checkCIF report

## Figures and Tables

**Table 1:** Hydrogen-bond geometry (Å, °)

*D*—H⋯*A*	*D*—H	H⋯*A*	*D*⋯*A*	*D*—H⋯*A*
O1—H1*O*1⋯O2	0.80	1.87	2.591 (3)	150
C11—H11*A*⋯O2^i^	0.93	2.53	3.416 (4)	160

Symmetry code: (i) 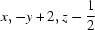.

**Fig. 1.**
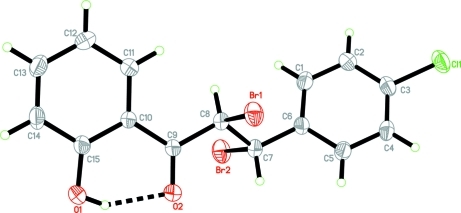
The molecular structure of the title compound, showing 30% probability displacement ellipsoids. The dashed line shows an intramolecular hydrogen bond.

**Fig. 2.**
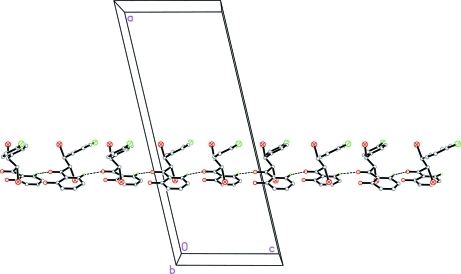
The crystal packing of the title compound, viewed along the b axis. Weak C—H···O hydrogen bonds are shown as dashed lines.

## supplementary crystallographic information

### Comment

For a structurally simple group of compounds, chalcones display an impressive array of biological activities, among which antimalarial (Liu *et al.*, 2003), antiprotozoal (Nielson *et al.*, 1998), nitric oxide inhibition (Rajas *et al.*, 2002) and anticancer activities (Dinkova-Kostova *et al.*, 1998) have been reported in the literature. Among several organic compounds reported for non-linear optical (NLO) properties, chalcone derivatives are notable materials for their excellent blue light transmittance and good crystallizability. They provide a necessary configuration to show NLO properties, with two planar rings connected through a conjugated double bond (Goto *et al.*, 1991; Uchida *et al.*, 1998; Tam *et al.*, 1989; Indira *et al.*, 2002; Sarojini *et al.*, 2006). The substitution of a bromo group on either of the phenyl rings can influence the non-centrosymmetric crystal packing. The bromo group can obviously improve the molecular first-order hyperpolarizabilities and can effectively reduce dipole-dipole interactions between the molecules. Chalcone derivatives usually have a lower melting temperature, which can be a drawback when we use these crystals in optical instruments. Chalcone dibromides usually have higher melting points and are thermally stable. Only a few structures of these compounds have been reported (Butcher, Yathirajan, Anilkumar *et al.*, 2006; Butcher, Yathirajan, Sarojini *et al.*,2006; Harrison *et al.*, 2005; Yathirajan, Mayekar *et al.*, 2007; Yathirajan, Vijesh *et al.*, 2007). In continuation to our studies on crystal structures of chalcones, we report the synthesis and crystal structure of the title compound.

In the title compound (Fig. 1), an *S*(6) ring motif (Bernstein *et al.*, 1995) is formed *via* the intramolecular O1—H1O1···O2 hydrogen bond (Table 1). The dihedral angle formed between the chloro-substituted benzene ring (C1–C6) and hydroxy-substituted benzene ring (C10–C15) is 34.10 (15)°.

In the crystal packing (Fig. 2), intermolecular C11—H11A···O2^i^ hydrogen bonds (Table 1) link the molecules into chains along the *c* axis.

### Experimental

(2*E*)-1-(2-Hydroxyphenyl)-3-(4-chlorophenyl)prop-2-en-1-one (0.01 mol) was treated with bromine in acetic acid (30%) until the orange colour of the solution persisted. After stirring for half an hour, the contents were poured onto crushed ice. The resulting solid mass was collected by filtration. The compound was dried and recrystallized from ethanol. Crystals suitable for structure determination were obtained from acetone by slow evaporation (*m. p.* = 395–397 K). Composition: Found (Calculated) for C_15_H_11_Br_2_ClO_2_, C: 43.19 (43.05); H: 2.68 (2.65).

### Refinement

H1O1 was located in a difference Fourier map and was fixed in its found position with *U*_iso_(H) = 1.5 *U*_eq_(O) [O–H = 0.7971 Å]. The remaining H atoms were positioned geometrically and refined using the riding model with *U*_iso_(H) = 1.2 or 1.5 *U*_eq_(C) [C–H = 0.93 to 0.98 Å]. Seven outliners were omitted for the final refinement, -22 0 2, -21 1 2, -9 1 1, -20 0 2, 1 1 0, 2 0 0 and -5 1 8.

### Crystal data

**Table:**

C_15_H_11_Br_2_ClO_2_	*F*(000) = 1632
*M**_r_* = 418.51	*D*_x_ = 1.860 Mg m^−^^3^
Monoclinic, *C*2/*c*	Mo *K*α radiation, λ = 0.71073 Å
Hall symbol: -C 2yc	Cell parameters from 4431 reflections
*a* = 29.075 (3) Å	θ = 2.9–29.1°
*b* = 9.2358 (10) Å	µ = 5.60 mm^−^^1^
*c* = 11.4374 (12) Å	*T* = 297 K
β = 103.290 (2)°	Block, yellow
*V* = 2989.0 (6) Å^3^	0.39 × 0.36 × 0.22 mm
*Z* = 8	

### Data collection

**Table:**

Bruker SMART APEXII DUO CCD area-detector diffractometer	5375 independent reflections
Radiation source: fine-focus sealed tube	3337 reflections with *I* > 2σ(*I*)
graphite	*R*_int_ = 0.034
φ and ω scans	θ_max_ = 32.6°, θ_min_ = 2.9°
Absorption correction: multi-scan (*SADABS*; Bruker, 2009)	*h* = −43→39
*T*_min_ = 0.218, *T*_max_ = 0.379	*k* = −13→13
16663 measured reflections	*l* = −13→17

### Refinement

**Table:**

Refinement on *F*^2^	Primary atom site location: structure-invariant direct methods
Least-squares matrix: full	Secondary atom site location: difference Fourier map
*R*[*F*^2^ > 2σ(*F*^2^)] = 0.037	Hydrogen site location: inferred from neighbouring sites
*wR*(*F*^2^) = 0.122	H-atom parameters constrained
*S* = 1.04	*w* = 1/[σ^2^(*F*_o_^2^) + (0.0657*P*)^2^] where *P* = (*F*_o_^2^ + 2*F*_c_^2^)/3
5375 reflections	(Δ/σ)_max_ = 0.001
181 parameters	Δρ_max_ = 0.61 e Å^−^^3^
0 restraints	Δρ_min_ = −0.47 e Å^−^^3^

### Special details

**Table:**

Geometry. All e.s.d.'s (except the e.s.d. in the dihedral angle between two l.s. planes) are estimated using the full covariance matrix. The cell e.s.d.'s are taken into account individually in the estimation of e.s.d.'s in distances, angles and torsion angles; correlations between e.s.d.'s in cell parameters are only used when they are defined by crystal symmetry. An approximate (isotropic) treatment of cell e.s.d.'s is used for estimating e.s.d.'s involving l.s. planes.
Refinement. Refinement of *F*^2^ against ALL reflections. The weighted *R*-factor *wR* and goodness of fit *S* are based on *F*^2^, conventional *R*-factors *R* are based on *F*, with *F* set to zero for negative *F*^2^. The threshold expression of *F*^2^ > σ(*F*^2^) is used only for calculating *R*-factors(gt) *etc*. and is not relevant to the choice of reflections for refinement. *R*-factors based on *F*^2^ are statistically about twice as large as those based on *F*, and *R*- factors based on ALL data will be even larger.

### Fractional atomic coordinates and isotropic or equivalent isotropic displacement parameters (Å^2^)

**Table:**

	*x*	*y*	*z*	*U*_iso_*/*U*_eq_	
Cl1	0.04064 (3)	0.29705 (9)	0.14615 (9)	0.0677 (2)	
Br1	0.191164 (10)	0.81997 (3)	0.36623 (3)	0.05335 (11)	
Br2	0.046011 (11)	0.98145 (4)	0.41259 (3)	0.06342 (12)	
O1	0.19186 (10)	1.3218 (2)	0.57890 (18)	0.0611 (6)	
H1O1	0.1812	1.2476	0.5965	0.092*	
O2	0.15388 (9)	1.0666 (2)	0.55101 (17)	0.0574 (5)	
C1	0.06311 (12)	0.7159 (3)	0.2247 (3)	0.0545 (7)	
H1A	0.0577	0.8040	0.1845	0.065*	
C2	0.04955 (11)	0.5874 (3)	0.1622 (3)	0.0553 (7)	
H2A	0.0355	0.5891	0.0805	0.066*	
C3	0.05732 (10)	0.4580 (3)	0.2236 (3)	0.0480 (6)	
C4	0.07828 (10)	0.4529 (3)	0.3435 (3)	0.0480 (6)	
H4A	0.0835	0.3645	0.3832	0.058*	
C5	0.09170 (10)	0.5816 (3)	0.4055 (3)	0.0469 (6)	
H5A	0.1056	0.5791	0.4873	0.056*	
C6	0.08443 (9)	0.7138 (3)	0.3460 (2)	0.0422 (5)	
C7	0.10038 (10)	0.8494 (3)	0.4158 (3)	0.0444 (6)	
H7A	0.1148	0.8237	0.4993	0.053*	
C8	0.13502 (9)	0.9403 (3)	0.3658 (2)	0.0417 (5)	
H8A	0.1204	0.9719	0.2839	0.050*	
C9	0.15471 (10)	1.0703 (3)	0.4438 (2)	0.0421 (5)	
C10	0.17415 (9)	1.1927 (2)	0.3898 (2)	0.0376 (5)	
C11	0.17591 (11)	1.1946 (3)	0.2682 (2)	0.0481 (6)	
H11A	0.1641	1.1165	0.2191	0.058*	
C12	0.19496 (12)	1.3112 (3)	0.2210 (3)	0.0550 (7)	
H12A	0.1959	1.3116	0.1403	0.066*	
C13	0.21269 (11)	1.4279 (3)	0.2937 (3)	0.0545 (7)	
H13A	0.2253	1.5066	0.2611	0.065*	
C14	0.21187 (10)	1.4284 (3)	0.4115 (3)	0.0516 (7)	
H14A	0.2242	1.5071	0.4593	0.062*	
C15	0.19263 (10)	1.3118 (3)	0.4619 (2)	0.0428 (5)	

### Atomic displacement parameters (Å^2^)

**Table:**

	*U*^11^	*U*^22^	*U*^33^	*U*^12^	*U*^13^	*U*^23^
Cl1	0.0831 (6)	0.0495 (4)	0.0769 (5)	−0.0197 (4)	0.0312 (4)	−0.0233 (4)
Br1	0.05194 (17)	0.04197 (15)	0.0691 (2)	0.00312 (11)	0.02003 (14)	−0.00637 (12)
Br2	0.05503 (19)	0.05123 (18)	0.0874 (3)	0.00816 (13)	0.02336 (16)	−0.00960 (15)
O1	0.0985 (17)	0.0433 (10)	0.0428 (11)	−0.0141 (10)	0.0186 (11)	−0.0098 (8)
O2	0.0898 (16)	0.0454 (10)	0.0388 (10)	−0.0148 (10)	0.0190 (10)	−0.0032 (8)
C1	0.0681 (18)	0.0395 (13)	0.0523 (17)	−0.0053 (13)	0.0062 (14)	0.0032 (12)
C2	0.0684 (18)	0.0489 (15)	0.0468 (16)	−0.0067 (13)	0.0092 (13)	−0.0065 (12)
C3	0.0495 (14)	0.0417 (13)	0.0570 (17)	−0.0074 (11)	0.0206 (12)	−0.0129 (12)
C4	0.0531 (15)	0.0315 (11)	0.0615 (18)	−0.0039 (10)	0.0174 (13)	0.0012 (11)
C5	0.0507 (14)	0.0390 (12)	0.0507 (15)	−0.0021 (11)	0.0111 (11)	0.0028 (11)
C6	0.0475 (13)	0.0345 (11)	0.0454 (14)	−0.0047 (10)	0.0121 (11)	−0.0022 (10)
C7	0.0536 (14)	0.0328 (11)	0.0476 (14)	−0.0016 (10)	0.0132 (11)	0.0000 (10)
C8	0.0507 (13)	0.0330 (11)	0.0418 (13)	−0.0013 (10)	0.0118 (11)	−0.0011 (9)
C9	0.0561 (14)	0.0320 (10)	0.0365 (13)	−0.0020 (10)	0.0076 (11)	−0.0007 (9)
C10	0.0464 (12)	0.0305 (10)	0.0354 (12)	−0.0008 (9)	0.0083 (10)	−0.0001 (9)
C11	0.0611 (16)	0.0437 (13)	0.0388 (14)	−0.0058 (12)	0.0100 (12)	−0.0031 (10)
C12	0.0691 (18)	0.0549 (16)	0.0433 (15)	−0.0072 (14)	0.0178 (13)	0.0084 (12)
C13	0.0631 (17)	0.0412 (13)	0.0581 (18)	−0.0073 (12)	0.0119 (14)	0.0105 (12)
C14	0.0586 (16)	0.0306 (11)	0.0623 (18)	−0.0067 (11)	0.0077 (13)	−0.0024 (11)
C15	0.0513 (14)	0.0319 (11)	0.0432 (14)	0.0005 (10)	0.0065 (11)	−0.0002 (9)

### Geometric parameters (Å, °)

**Table:**

Cl1—C3	1.741 (3)	C6—C7	1.501 (3)
Br1—C8	1.974 (3)	C7—C8	1.520 (4)
Br2—C7	1.990 (3)	C7—H7A	0.9800
O1—C15	1.347 (3)	C8—C9	1.527 (3)
O1—H1O1	0.7971	C8—H8A	0.9800
O2—C9	1.232 (3)	C9—C10	1.463 (3)
C1—C6	1.383 (4)	C10—C11	1.403 (4)
C1—C2	1.395 (4)	C10—C15	1.406 (3)
C1—H1A	0.9300	C11—C12	1.376 (4)
C2—C3	1.378 (4)	C11—H11A	0.9300
C2—H2A	0.9300	C12—C13	1.386 (4)
C3—C4	1.367 (4)	C12—H12A	0.9300
C4—C5	1.393 (4)	C13—C14	1.353 (4)
C4—H4A	0.9300	C13—H13A	0.9300
C5—C6	1.390 (4)	C14—C15	1.398 (4)
C5—H5A	0.9300	C14—H14A	0.9300
			
C15—O1—H1O1	106.8	C7—C8—Br1	107.91 (17)
C6—C1—C2	120.7 (3)	C9—C8—Br1	104.04 (17)
C6—C1—H1A	119.6	C7—C8—H8A	110.2
C2—C1—H1A	119.6	C9—C8—H8A	110.2
C3—C2—C1	118.9 (3)	Br1—C8—H8A	110.2
C3—C2—H2A	120.6	O2—C9—C10	122.7 (2)
C1—C2—H2A	120.6	O2—C9—C8	118.0 (2)
C4—C3—C2	121.6 (2)	C10—C9—C8	119.3 (2)
C4—C3—Cl1	119.2 (2)	C11—C10—C15	118.4 (2)
C2—C3—Cl1	119.2 (2)	C11—C10—C9	122.3 (2)
C3—C4—C5	119.3 (3)	C15—C10—C9	119.3 (2)
C3—C4—H4A	120.3	C12—C11—C10	120.6 (3)
C5—C4—H4A	120.3	C12—C11—H11A	119.7
C6—C5—C4	120.4 (3)	C10—C11—H11A	119.7
C6—C5—H5A	119.8	C11—C12—C13	120.0 (3)
C4—C5—H5A	119.8	C11—C12—H12A	120.0
C1—C6—C5	119.1 (2)	C13—C12—H12A	120.0
C1—C6—C7	122.3 (2)	C14—C13—C12	120.7 (3)
C5—C6—C7	118.6 (2)	C14—C13—H13A	119.6
C6—C7—C8	114.2 (2)	C12—C13—H13A	119.6
C6—C7—Br2	110.76 (19)	C13—C14—C15	120.5 (3)
C8—C7—Br2	104.30 (16)	C13—C14—H14A	119.7
C6—C7—H7A	109.1	C15—C14—H14A	119.7
C8—C7—H7A	109.1	O1—C15—C14	117.2 (2)
Br2—C7—H7A	109.1	O1—C15—C10	123.0 (2)
C7—C8—C9	113.9 (2)	C14—C15—C10	119.7 (3)
			
C6—C1—C2—C3	0.7 (5)	Br1—C8—C9—O2	−94.9 (3)
C1—C2—C3—C4	−0.6 (5)	C7—C8—C9—C10	−158.4 (2)
C1—C2—C3—Cl1	−179.8 (3)	Br1—C8—C9—C10	84.4 (2)
C2—C3—C4—C5	0.7 (4)	O2—C9—C10—C11	178.6 (3)
Cl1—C3—C4—C5	179.9 (2)	C8—C9—C10—C11	−0.7 (4)
C3—C4—C5—C6	−0.8 (4)	O2—C9—C10—C15	−0.2 (4)
C2—C1—C6—C5	−0.8 (5)	C8—C9—C10—C15	−179.5 (2)
C2—C1—C6—C7	178.8 (3)	C15—C10—C11—C12	−0.5 (4)
C4—C5—C6—C1	0.9 (4)	C9—C10—C11—C12	−179.3 (3)
C4—C5—C6—C7	−178.7 (3)	C10—C11—C12—C13	0.1 (5)
C1—C6—C7—C8	−56.8 (4)	C11—C12—C13—C14	0.5 (5)
C5—C6—C7—C8	122.8 (3)	C12—C13—C14—C15	−0.7 (5)
C1—C6—C7—Br2	60.6 (3)	C13—C14—C15—O1	−178.2 (3)
C5—C6—C7—Br2	−119.8 (2)	C13—C14—C15—C10	0.3 (4)
C6—C7—C8—C9	−174.5 (2)	C11—C10—C15—O1	178.7 (3)
Br2—C7—C8—C9	64.5 (2)	C9—C10—C15—O1	−2.5 (4)
C6—C7—C8—Br1	−59.5 (3)	C11—C10—C15—C14	0.3 (4)
Br2—C7—C8—Br1	179.45 (11)	C9—C10—C15—C14	179.2 (3)
C7—C8—C9—O2	22.3 (4)		

### Hydrogen-bond geometry (Å, °)

**Table:**

*D*—H···*A*	*D*—H	H···*A*	*D*···*A*	*D*—H···*A*
O1—H1O1···O2	0.80	1.87	2.591 (3)	150
C11—H11A···O2^i^	0.93	2.53	3.416 (4)	160.

Symmetry codes: (i) *x*, −*y*+2, *z*−1/2.
